# Eutectic Nano-Droplet Template Injection into Bulk Silicon to Construct Porous Frameworks with Concomitant Conformal Coating as Anodes for Li-Ion Batteries

**DOI:** 10.1038/srep10381

**Published:** 2015-05-19

**Authors:** Fei Qu, Chilin Li, Zumin Wang, Yuren Wen, Gunther Richter, Horst P. Strunk

**Affiliations:** 1 State Key Laboratory of High Performance Ceramics and Superfine Microstructure, Shanghai Institute of Ceramics, Chinese Academy of Sciences, Shanghai 200050, China; 2 Institute for Materials Science, Chair of Materials Physics, University of Stuttgart, Heisenbergstr. 3, 70569 Stuttgart, Germany; 3 Max Planck Institute for Intelligent Systems (formerly Max Planck Institute for Metals Research), Heisenbergstr. 3, 70569 Stuttgart, Germany

## Abstract

Building porosity in monolithic materials is highly desired to design 3D electrodes, however *ex-situ* introduction or *in-situ* generation of nano-scale sacrificial template is still a great challenge. Here Al-Si eutectic droplet templates are uniformly injected into bulk Si through Al-induced solid-solid convection to construct a highly porous Si framework. This process is concomitant with process-inherent conformal coating of ion-conductive oxide. Such an *all-in-one* method has generated a (continuously processed) high-capacity Si anode integrating longevity and stable electrolyte-anode diaphragm for Li-ion batteries (e.g. a reversible capacity as large as ~1800 mAh/g or ~350 μAh/cm^2^-μm with a CE of ~99% at 0.1 C after long-term 400 cycles).

Due to its highest theoretical capacity of 4200 mAh/g, Si is one of the most promising anode candidates for high energy Li ion batteries (LIBs)^1^,^2^. Application however faces two essential difficulties. Firstly, discharging and charging (lithiation/delithiation) processes lead to the formation and decomposition of Li_22_Si_5_ phase inclusions that suffer from serious volume expansion/contraction of up to 400%,^1^,^2^ which disappointingly results in bulk Si electrode cracking and pulverization followed by electrical contact loss during long term battery cycling ('breathing'). Secondly, a so-called solid electrolyte interphase (SEI) forms and accumulates as a consequence of a chemical reaction between electrolyte and Si that degrades capacity, reversibility or coulombic efficiency (CE) of LIBs^1^,^2^. To overcome the material fatigue caused by the formation of Li-rich alloy phases (e.g. Li_22_Si_5_), the nanoscale and porous structures of Si are highly desired^3^,^4^,^5^,^6^. However, undecorated nanostructures always allow the more exposure of Si surfaces to electrolyte and thus the formation of thicker SEI. The degradation due to SEI formation can be counteracted by the introduction of a thin layer, such as a suitable polymer film,^5^ that chemically separates electrolyte and Si anode but is transparent to Li-ions. Atomic layer deposition (ALD) as one of the most popular coating approaches has been widely used to deposit a conformal nano-scale Al oxide layer on nanostructured Si^7^,^8^. Unfortunately, this complicated auxiliary process is out of the consideration for the large-scale LIB industry owing to its low production efficiency and high cost. Thus, exploring a synthesis of nano-structure with a process-inherent coating is still highly required for pursuing high-performance Si anodes.

Si films attached a substrate have the additional advantages that they do not require the extra inactive binders (e.g. PVDF) and conductive substances (e.g. carbon black), and could therefore achieve the near-theoretical capacity^9^,^10^,^11^. As a remedy for overcoming the 'breathing action', the use of Si films with theoretically a thickness of less than ~300 nm that may freely breath into the thickness dimension during cycling has been considered^12^,^13^. For the thicker films, a great challenge remains in the utilization ratio of active materials, since serious capacity fading is often met after several cycles owing to film delamination or the difficult transport of Li-ions/electrons to the deep bulk of thick films^13^. Further improvement thereby requires the introduction of 2- to 3-dimensional (3D) micrometer-structured Si films (which can overcome the thickness limit of ~300 nm). All this leads up to the introduction of nano-architectures into micro-sized Si films. They are of significant importance in terms of the construction of high-aspect-ratio electrodes and 3D all-solid-state LIBs^14^,^15^. Such structured silicon layers may even add to achieve a much higher area- or volume-specific electric capacity. However most the chemical or physical vapor deposition methods (e.g. template-array assisted or glancing angle deposition) appear to be unsuitable to produce large-scale Si thick films meantime containing well-defined nano-architecture^9^,^10^,^11^.

One of the methods to design 3D electrodes is to build porosity in high-density monolithic materials. However how to *ex-situ* introduce or *in-situ* generate sacrificial template (especially in nano-scale) evenly in bulk phase is still a great challenge. In the present work we utilize a principle of aluminum induced crystallization (AIC) and solid-solid convective transport between Al and Si to thermally drive an *ex-situ* injection of Al-Si eutectic droplets into bulk Si^16^,^17^,^18^,^19^. Fortunately, these droplets are Al-rich, nano-sized and uniformly distributed in Si matrix, and therefore can serve as sacrificial templates to construct a highly porous Si framework film with improved charge carrier conductivity. This easy-to-apply procedure is based on a fast eutectic reaction between a very thin Al film (nano-sized thickness) and a thick amorphous Si (a-Si) film (thickness ~1500 nm). Melting of diffusible Al is necessary to form eutectic fluid phase, which penetrations into the whole Si and stays as confined droplet-like templates. For battery application, such a porosity can effectively accommodate volume changes and improve electrolyte infiltration. In addition, Al-induced crystallization of a-Si (likely with concomitant heavy p-doping of the crystallized Si by Al) can increase electron conductivity across the whole thick film. After removal of the Al by etching, a nanometer thick layer of Al oxide remains on all surfaces of the Si forming a conformal stable artificial SEI. This layer guarantees a high columbic efficiency during long-term cycling. Such an *all-in-one* method has generated the Si thick films with its desired high porosity and conformal coating, which demonstrate excellent Li-storage performance (e.g. a reversible capacity as large as ~1800 mAh/g or ~350 μAh/cm^2^-μm with a CE of ~99% at 0.1 C after long-term 400 cycles) and show their potential as 3D anodes for high-energy solid LIBs.

## Results

### Preparation and characterization of Si porous framework

**Figure 1:**
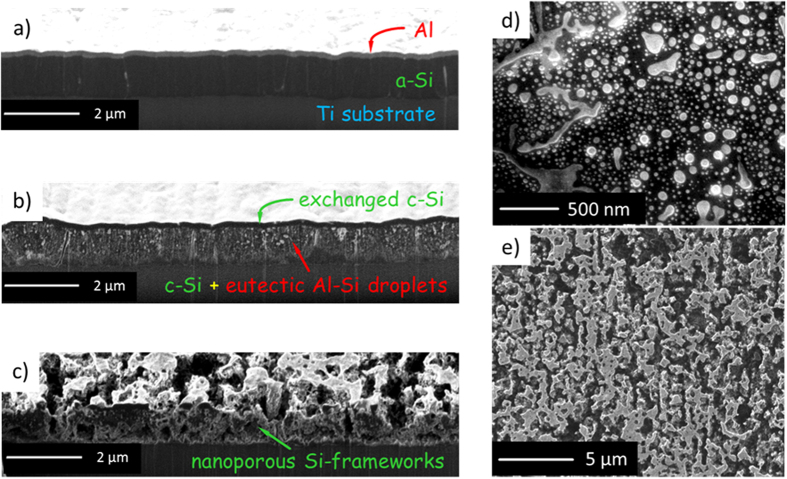
Morphology and microstructure evolution during constructing Si porous framework. (**a-c**) Cross-sectional FIB images of microstructure evolution in Al/Si bilayer samples with a thickness ratio of Si:Al** **=** **10:1: (**a**) as-deposited Al/a-Si bilayer, (**b**) after annealing at 600** **°C for 30** **min: upper layer: continuous exchanged crystalline Si (c-Si), lower layer: eutectic Al-Si droplets in bright area and c-Si in dark area, (**c**) after additional etching of the annealed sample: porous c-Si frameworks. (**d**) Top view SEM image of the lower layer after annealing (bright area: eutectic Al-Si precipitates, dark area: c-Si). For taking such images, the sample was sputtered by Ga ion beam in a FIB microscope to remove the upper layer away. (**e**) Top view secondary electron image of porous Si framework formed after etching of the annealed sample by alkali solution.

**Figure 2:**
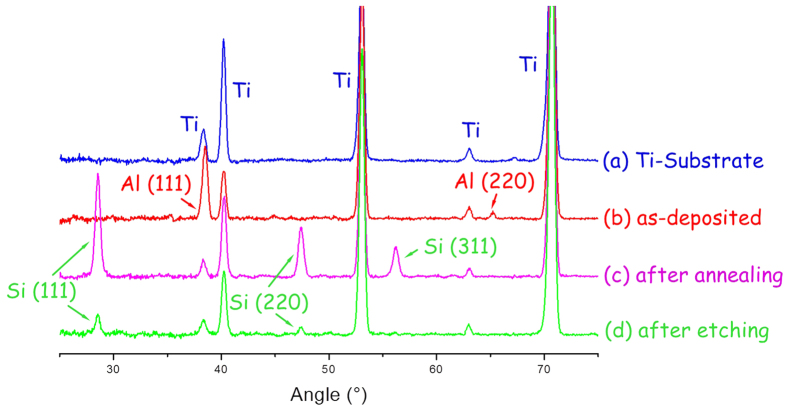
Crystallization characterization of different fabrication stages. XRD patterns of Al/Si bilayer samples with a thickness ratio of Si:Al = 10:1 at different annealing/etching stages: (**a**) Ti-substrate as a reference (blue line), (**b**) as-deposited Al/a-Si bilayer (red line), (**c**) the sample after annealing at 600 °C for 30 min followed by rapid cooling (< 2 min) to room temperature (purple line), (**d**) the sample after additional etching (green line).

The Si porous framework is in this work prepared as thick film by starting with the deposition in a high-vacuum multi-target direct-current (DC) sputtering system of an Al/Si bilayer with asymmetric sublayer thicknesses (i.e.1500 nm for Si and 150 nm for Al). A polished Ti foil substrate kept at room temperature serves as substrate. A cross-sectional focused-ion-beam (FIB) image of as-deposited film specimen is shown in Fig. 1a. The thin Al overlayer (brighter area) and the Si bottom layer (darker area) can be clearly discerned. The corresponding X-ray diffraction (XRD) pattern in Fig. 2b is characterized by diffraction peaks corresponding only to polycrystalline Al (plus substrate signals as seen from Fig. 2a). The lack of any Si diffraction peaks confirms the amorphous structure of Si sublayer. The specimen was then annealed for 30 min in a vacuum oven at 600 °C, i.e. slightly above the eutectic point (577 °C) of Al-Si system, followed by a fast cooling within 2 min to room temperature (RT). After annealing, a nearly continuous Si film (dark area) with a thickness of about 150 nm has formed at the original location of Al overlayer. Only a small part of the whole Si has been exchanged this way. The Al exists mainly in a large amount of small droplet-like precipitates (bright spots) within the original Si sublayer (see the cross-sectional FIB image of Fig. 1b and the top view scanning electron microscopy (SEM) image of Fig. 1d). The size distribution of bright Al-rich droplets ranges from several nanometer to hundreds of nanometer. Some of the droplets come in contact with each other to form much larger aggregates. The X-ray diffraction now reveals, in addition to the Ti- and Al-peaks, also Si-peaks (111, 220, 311, see Fig. 2c) indicating the Si has crystallized. Since crystallization of pure a-Si normally occurs at ~700 °C, this crystallization process occurring at lower temperature can be ascribed to the AIC mechanism where the a-Si- and Al-layers exchange their locations by solid state convective transport and the Si layer simultaneously crystallizes^18^,^19^. Si crystallization occurs also adjacent to the Al-rich droplets and because of the quasi-uniform distribution of droplets, most of the Si has crystallized. As last steps, the annealed specimen was rinsed in 1 mol/L NaOH solution to selectively etch off the droplet-like Al-rich precipitates, flushed with distilled water and finally dried with heated Ar. Consequently, a porous Si framework with nanocrystalline feature remains as shown in the cross-sectional FIB and top-viewing secondary electron images in Fig. 1c,1e and the corresponding XRD pattern in Fig. 2d.

**Figure 3:**
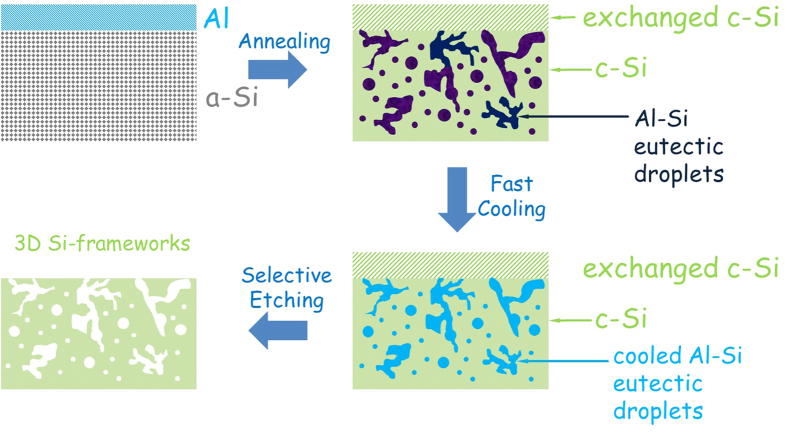
Scheme of annealing/etching steps to construct Si porous framework from Al/Si bilayers. Procedure to fabricate Si frameworks with porous structure from Al/Si bilayers with a thickness ratio of Si:Al** = **10:1. The Al/a-Si bilayer is firstly annealed at 600** **^o^C to form a Si matrix with Al-rich eutectic droplets embedded inside and with a thin layer of c-Si exchanged to the top zone. During fast cooling to room temperature, the Al-Si eutectic droplets solidify while preserving their original shape without phase separation and recrystallization (keeping the droplets amorphous). During the etching process, the Al-rich eutectic droplets as well as the top c-Si layer are removed, leaving the desired 3D porous framework of c-Si.

Figure 3 summarizes all these processing steps required to prepare a Si thick film with porous framework structure. It is known that the AIC process (and solid-solid convection) starts at such a low temperature as ~200 ^o^C^20^. Therefore, our annealing temperature (600 ^o^C) is high enough to complete a fast and thorough exchange between Al and Si layers that leads to a continuous Si layer at the original location of Al overlayer^19^,^21^. A magnified cross-sectional FIB image of the annealed specimen as shown in Figure S1 further confirms the continuity of this exchanged Si overlayer. Since the annealing temperature is higher than the eutectic point (577 ^o^C) of Al-Si, the Al material diffusing downwards would melt and form a eutectic with about 13 at.% Si (at 600 ^o^C) according to the binary alloy phase diagram of Al-Si (see Figure S2)^22^. This Al-rich alloying phase is present as nano-sized droplets and distributed throughout the Si sublayer as sketched in Fig. 3. A very thin thickness of Al layer appears to be crucial to construct discrete, uniform, small droplets. The termination of annealing is followed by a fast cooling of the specimen to rapidly solidify these eutectic droplets. This fast cooling is expected to enable the preserving of the shape of droplets without recrystallization and phase separation. Indeed, the diffraction peaks corresponding to crystalline Al have disappeared after cooling to RT (see Fig. 2c), indicating an amorphous alloy state of cooled Al-Si eutectic droplets. During etching off the Al-rich droplets, a large part of the crystallized Si overlayer was washed away under the effect of residual stresses^18^. The less pronounced diffraction peaks of Si observable after etching imply the (nano)crystalline structure of the formed porous Si frameworks (Fig. 2d).

**Figure 4:**
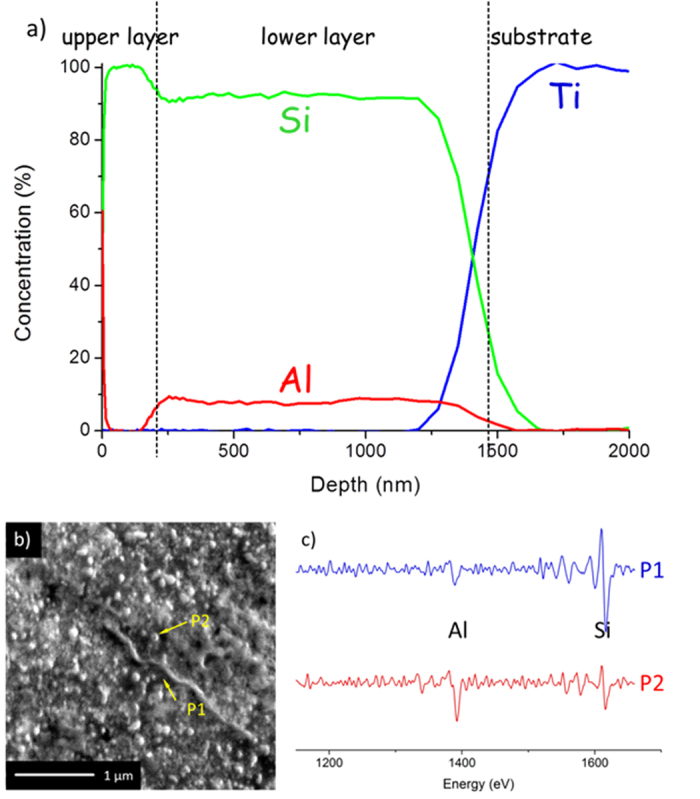
Surfaceand bulk component analysis of annealed Al/Si bilayer by AES. (**a**) Molar concentration depth profile of the Al/Si bilayer sample after annealing by Auger electron spectroscopy (AES). (**b**) surface secondary electron image of the lower layer with AES spots at different area (P1 at the dark area, P2 at the bright area). The sample was ion-etched with Ga ions to remove the upper layer. (**c**) AES analysis from P1 and P2. For P1 a strong Si signal is seen and the Al signal is very weak, whereas for P2 both the signals are pronounced.

Auger electron spectroscopy (AES) concentration-depth profiles in Fig. 4a confirm the almost complete exchange of the Al layer by a Si layer, with the observation of Si to ~100 at.% reside at the top in a thickness of ~150 nm. A nearly constant Al molar concentration of about 10% is observed across the lower layer zone with about 90 at.% Si, indicating the uniform distribution of the formed Al-rich droplets in the Si matrix. The local AES analysis in combination with secondary electron image on the lower layer zone is shown in Fig. 4b,3c. Evidently, the dark matrix region (P1 in Fig. 4b) corresponds to almost pure Si, whereas the bright droplet region (P2 in Fig. 4b) contains both Al and Si, and is Al-rich. The interface between the lower layer and the Ti substrate appears to be broad in the AES depth profile which suggests that certain interdiffusion of Si and Ti has occurred during the annealing with possible formation of interfacial Ti silicides^23^, which are very likely amorphous because of lack of respective X-ray diffraction peaks (Fig. 2c). Such interface reactions could promote close contact of the Si anode material with the substrate surface, suppress delamination, facilitate electron transport to substrate, and hence contribute to the electrochemical performance as discussed below.

**Figure 5:**
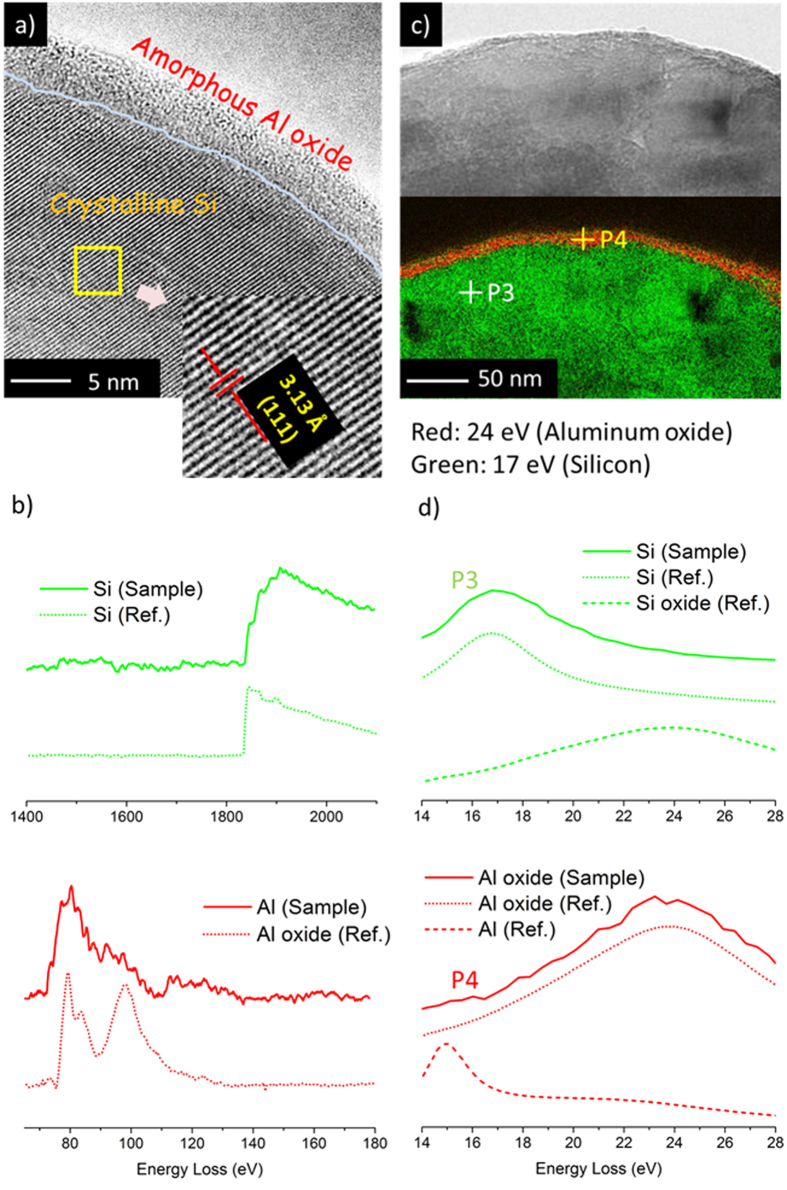
Component and structure analysis of surface and bulk of Si framework by TEM and EELS. (**a**) HRTEM image of the specimen after etching: an amorphous layer formed on c-Si surface (the introduced pale blue line traces the approximate crystalline-amorphous interface). (**b**) EELS core-loss spectra of the same specimen (red solid line for Al and green for Si). The reference spectra (dotted lines) of Al and Si components are included: Al L_23_-edge spectrum without background (red) and Si K-edge spectrum without background (green). (**c**) Bright-field image (above) and overlaid color-coded (red for Al oxide and green for Si) EFTEM mapping (below) based on plasmon peak position (see d). (**d**) EELS low-loss spectra taken from area marked by the dots P3 (green curve) and P4 (red curve). The reference spectra of Al, Si and their oxides are also labeled for comparison (referring to Ref. 24).

The Si based porous frameworks can be analyzed regarding its microstructure and composition by high-resolution transmission electron microscopy (HRTEM) and electron energy-loss spectroscopy (EELS) (see Fig. 5). Fig. 5a shows a HRTEM cross-section through the porous framework, and the crystalline Si is discernable from the lattice fringe contrast with interdistance of 3.13 Å characteristic of Si(111) lattice planes. An amorphous nanoscale layer approximately 5 nm thick, consisting of amorphous Al oxide (as will be seen in a minute), covers this crystalline volume quasi-conformally. From the EELS core-loss spectra (solid line in Fig. 5b), both the Al and Si signals are detected in the specimen based on the standard values referred to Ref. 24 (corresponding dotted line)^24^. However Al, Si and their oxides can be hardly distinguished from each other in the core-loss spectra. In order to discriminate the chemical bond status of Al and Si we resort to the EFTEM (energy-filtering transmission electron microscopy) mapping at the low-loss EELS spectra (Fig. 5d)^25^. Such low-loss EELS spectra have been used for elemental mapping and the lower micrograph of Fig. 5c shows a respective color-coded overlay of the pixel-wise analysis (for procedure see Experimental Section). Inside the specimen the peak at almost every pixel (e.g. P3 in Fig. 5c) locates at 17 eV in the low-loss EELS spectra (green solid curve in Fig. 5d). It is the characteristic plasmon peak position of pure Si (green dotted curve in Fig. 5d), which is quite different from its oxide (green dashed curve in Fig. 5d) and also different from Al and its oxide (red dotted and dashed curves in Fig. 5d). Combined with the HRTEM image (Fig. 5a), it indicates that the green region in Fig. 5c consists of crystalline Si. Consequently, the Al signal as observed in the core-loss spectra (red solid curve in Fig. 5b) can only result from the outer region of the samples. The spectra at the outer region (e.g. P4 in Fig. 5c and red solid curve in Fig. 5d) have an obvious maximum at 24 eV, in accordance with the reference values of Al and Si oxides (red dotted and green dashed lines in Fig. 5d). It is different from the signal of pure Al or Si with a peak at 15 eV or 17 eV (red dashed or green dotted line in Fig. 5d). Thus, the existence of a thin conformal oxide coating on the Si framework with about 5 nm in thickness is experimentally proven as represented by the red region in Fig. 5c. Because the oxidative etching of Al in NaOH solution is exothermic, the local heat release could promote local diffusion at the interface between Si matrix and Al-rich droplet^16^. Furthermore, during the etching process, aluminate precipitates are reported to form as a result of the reaction between Al and NaOH, and convert to alumina through ion exchange with carbon dioxide in air^26^. Intuitionally, the higher annealing temperature than eutectic one is also beneficial to the interaction of Al and Si beyond solubility limitation. On the other hand, the Si layer has not been exposed to air before growing the Al layer and therefore a substantial natural oxidation of surface Si can be ruled out. Based on the comprehensive analysis above, the coating component is dominated by amorphous Al oxide rather than Si oxide. Besides the TEM-EELS analyses, the Al-contained coating (<2 wt.% of the framework Si content) is also confirmed by inductively coupled plasma (ICP) measurement (see Method Section for detail).

### Electrochemistry of Si porous framework as LIB anode

Now let us turn to the benefit this anode material offers to LIB performance. It is known that Al oxide may serve as an artificial SEI with exclusive Li-ion permeability to improve the CE of Si anodes^7^,^27^. Previous progress has mainly focused on the ALD method to achieve conformal thin Al oxide coating.^8^ However, the ALD method is procedurally complicated and uneconomical, limiting its application in large-scale decoration of materials. The present method to build porosity inside dense Si surprisingly and beneficially brings about an *in-situ* conformal Al oxide coating, which is much simpler and of much lower cost as compared to ALD. This concomitant coating avoids the direct contact of Si surface with electrolyte and is expected to be more stable than the electrochemistry-driven SEI usually forming on the bare Si surface. It can also serve as additional ion-wire as long as the Li-Al-O surface phase is generated during the first lithiation. Furthermore, the growth temperature as high as 600 ^o^C likely leads to heavily p-type doping of Si by Al at least near the surface (or subsurface) of the prepared Si frameworks^20^. It has been reported that the resistivity of heavily p-doped poly-Si (10^−1^ Ω cm) is twelve orders of magnitude lower than that of intrinsic a-Si or six orders of magnitude lower than that of intrinsic poly-Si^28^. Therefore, the heavily doped phase provided that it forms and percolates in the whole Si framework can serve as additional electron-wire, which is particularly helpful for charge transport in such a thick film electrode free of conductive carbon.

**Figure 6:**
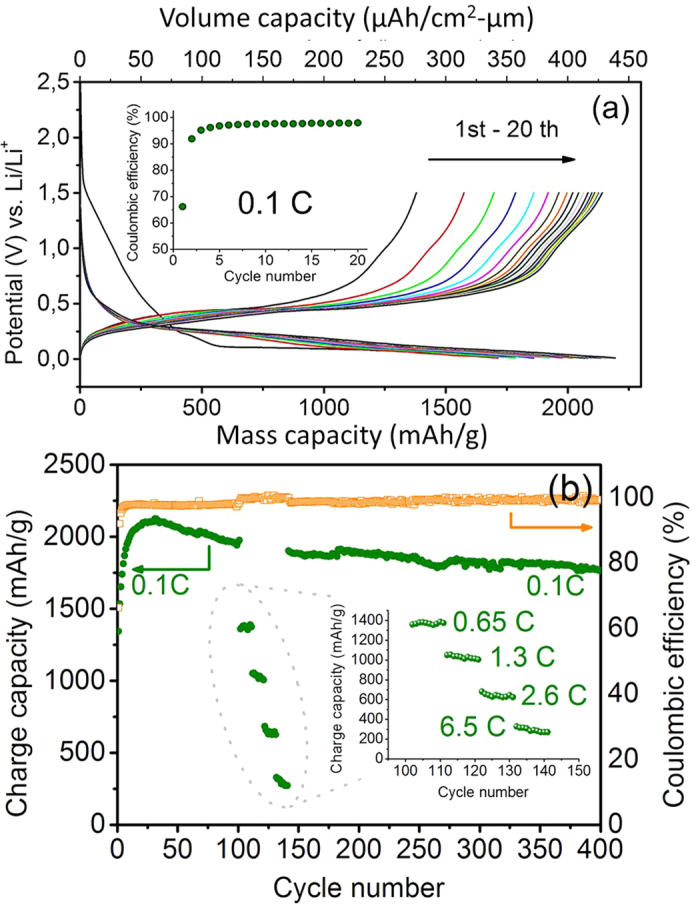
Li-storage performance of Si porous framework as anode. (**a**) Voltage vs m**a**ss-specific (lower abscissa) or volume-specific (upper abscissa) capacity profiles of nanoporous Si framework as anode at 0.1 C during the first twenty cycles in a voltage range of 0.01-1.5 V, inset of (**a**): The corresponding coulombic efficiency as a function of cycle number at 0.1 C within 20 cycles. (**b**) Charge capacity (green colored plots) or coulombic efficiency (orange colored plots) of nanoporous Si framework as a function of cycle number at a basic rates of 0.1 C under a long-term cycling up to 400 cycles. There is an intermediate cycle range (details in the inset) where the rate is changed stepwise from 0.1 C to 6.5 C and back to 0.1 C.

Figure 6 shows the galvanostatic performance of porous Si frameworks as measured by using additive-free electrolyte (for details see Experimental Section). Note that there is a sloped electrochemical curve with a substantial capacity of ~500 mAh/g during the first lithiation from 1.5 V to 0.1 V, where a typical plateau curve is followed until the ending of discharge (Fig. 6a). This characteristic plateau indicates the process of lithiation of poly-Si, and however disappears in the following discharge steps, which instead display sloped curves between 0.5 and 0.01 V owing to irreversible amorphization of poly-Si during the first cycling^29^. The charge processes always display sloped curves from 0.2 V to 0.7 V. One should find that both the capacity and CE are remarkably improved during the early cycling. As shown in Fig. 6a, the charge capacity is increased from ~1370 mAh/g to ~2150 mAh/g with the CE from 67% to 98% at 0.1 C in the first twenty cycles. This unusual electrochemical activation should be ascribed to the more exposure of original or newly formed Si surfaces to the more infiltrated electrolyte inside the porous framework. A reversible capacity of ~1780 mAh/g with a CE as high as 99% is still maintainable after a long-term 400 cycles (Fig. 6b). It is about 83% of the maximum capacity achieved at the 30^th^ cycle. Undoubtedly, the excellent cyclability benefits not only from the nanoporous structure for extra volume accommodation and strain release, but also from the conformal coating for less formation/accumulation of electrochemically driven SEIs. The reversible capacities of 1000 mAh/g and 600 mAh/g are also achievable at the higher rates of 1.2 C and 2.4 C respectively. After undergoing multi-cycling at as high as 6 C, the capacity at 0.1 C is still recoverable when the current density returns to 0.1 C after 150 cycles (Fig. 6b), indicating the robustness of Si framework structure under fast or long-term lithiation/delithiation. Indeed, the nano-porosity and interconnectivity of frameworks are well preserved after long-term galvanostatic test as confirmed from the SEM morphologies of cycled samples in Figure S3. As far as the quite large volume-specific capacity (e.g. ~430 μAh/cm^2^-μm at the 20^th^ cycle and ~350 μAh/cm^2^-μm at the 400^th^ cycle) is concerned, this Si porous framework is thought to be a potential 3D anode for all-solid-sate batteries as long as its pore regions can be partially filled by solid electrolyte components as ion-wires.

We also tested the similar annealing/etching steps on the Al-Si bilayer with the same individual thickness of 1500 nm for both the Al and Si sublayers. As shown in Figure S4, the layer exchange between Al and Si is initiated by AIC mechanism at the early annealing stage. However, under such a high annealing temperature, the diffused Al would melt with surrounding Si to form an eutectic alloy layer, which likely retards the further layer exchange. Indeed, we observe that most of Al remains at the top of specimen after annealing, meanwhile the lower part of the bottom Si layer with a thickness of ~1 μm is still compact. The intermixing between Al and Si in this case leads to an extremely uneven morphology with many sheet-shaped Si of micro-sized (~5 μm) width standing irregularly over the porous upper layer of Si after etching (also see Figure S5), which is unfavorable to its Li-storage electrochemistry. This result confirms the significance of thickness modulation of Al overlayer in the develop process.

## Discussion

The methods to prepare porous Si electrodes characterized by interconnected walls are still lacking. Recently, magnesiothermic reduction method and metal-assisted chemical etching have been adopted to construct porous Si from bulk SiO_x_ (x = 0-2)^6^,^30^,^31^. However these methods often required high temperature annealing (>650 ^o^C) or resorted to highly dangerous or corrosive agents (e.g. HF or H_2_O_2_). Complex redox reactions associated with Mg/Mg^2+^ or Ag/Ag^+^ have to occur. Our strategy can achieve etching (or porosity construction) at a lower annealing temperature without the occurrence of redox reaction. Furthermore the pores can propagate to deeper sites in bulk through solid-solid convection between layers by utilizing less reactive and expensive Al. Unlike the previous methods requiring a pre-precipitation of Ag or Mg nanoparticles in order to building well-defined porosity, in this work we merely want a simple deposition of continuous Al thin layer, which can automatically transform into discrete nano-droplet templates when melting with a small fraction of neighboring Si during convection. Several works also pursued a pre-alloying of Si-Al in order to arrange a uniform distribution of Al-template across the bulk^32^. In fact, this extra energy-consuming step is not necessary in view of strong permeation capability of Al-template triggered by AIC and solid-solid convection mechanism (taking effect at as low as 200 ^o^C)^20^. A relatively higher annealing temperature in our case is to guarantee the generation and separation of droplet-like templates. Another additional harvest from the use of Al-template is the concomitant formation of conformal Al-oxide coating, which has been widely accepted as one of the best candidates of artificial SEI^7^,^27^.

The bilayer architecture allows a sufficient contact between sacrificial template and active material, and is thought to be a good model to visualize the evolutions of microstructure and component depending on annealing time. After annealing/etching, this architecture itself can be considered as a potential 3D electrode for future flexible solid batteries, where the thickness and ratio of bilayer are tunable in order to obtain desired morphology and loading of active material (for a higher area- or volume-specific capacity). Film architecture is also a good reference and similar principles (e.g. metal-induced crystallization and solid-solid convection) can be extended to synthesize large-scale powder materials of porous alloys. Further directions will also focus on other textured substrates to further decrease the annealing temperature/duration and develop more regular nanoporous structures, as well as on more conductive alloy electrodes (e.g. Ge, which is expected to require a much lower annealing temperature) and on crystallization phenomena induced by other non-precious metals (e.g. Sn)^33^,^34^. As far as film growth technologies are concerned, it seems to be difficult for physical vapor deposition (PVD) methods to achieve sufficient nano-porosity especially when pursuing micron-above thickness of films. Post-deposition processing (e.g. solid-solid convection in our case) appears to provide new opportunity for PVD technologies on nanostructure construction.

In summary, a novel method based on *ex-situ* injection of Al-Si eutectic droplets as nano-sized templates is developed to prepare a highly porous Si framework in form of a micro-sized thick film that has advantageous properties as anode material for LIBs. This nanostructure is beneficial to electrolyte infiltration, volume accommodation and strain release during repeating lithiation. Besides as sacrificial templates for porosity, the Al components also serve as the sources for 5 nm thick conformal Al-oxide coating. The coating also acts as stable artificial SEI, which could effectively suppress the formation and accumulation of less stable electrochemistry-driven SEI. This *all-in-one* design by simultaneously creating porous nanostructure and conformal coating would strengthen the integrity and conductivity of electrode networks, leading as shown to a highly satisfactory retention of capacity as large as 1780 mAh/g with a CE of 99% within long-term 400 cycles. This method is inspiring to construct porous electrode configuration for volume-variable alloying or conversion materials as long as droplet-like or nano-dot templates can migrate into or be *in-situ* precipitated in the electroactive matrix. Such a Si porous film is also promising as 3D anode for all-solid-state batteries owing to its volume-specific capacity larger than 350 μAh/cm^2^-μm.

## Methods

### Sample preparation

Pure titanium foils (99.6%, Goodfellow) are used as substrates for sequentially depositing Si and Al layers. The substrates were rinsed with ethanol in ultrasonic cleaner to remove organic species on the surface. The bilayer samples were prepared by sputtering Si and Al targets in a home-made high vacuum multi-target DC sputtering system. After reaching a base vacuum pressure less than 1 × 10^−7^ mbar, the substrates were additionally cleaned with Ar^+^ ions for 1 min. Then a 1500 nm thick Si layer was deposited at room temperature by using a sputtering power of 100 W in an Ar atmosphere of 5.9 × 10^−3^ mbar, followed by depositing an Al layer at a power of 200 W in the same Ar pressure. To check the Al-thickness dependent morphology evolution, we intentionally chose two extremely different deposition thicknesses of Al: 150 nm (1/10 of the Si sublayer thickness) and 1500 nm (same as the Si sublayer thickness). The as-deposited Si/Al bilayers were then annealed in a vacuum oven at 600 °C for 30 min. After the samples were fast cooled down to room temperature within 2 min by blowing with Ar gas, they were further etched by 1 mol/L NaOH solution to remove Al selectively. Finally they were flushed with distilled water and dried with heated Ar. The mass of final porous Si framework films is obtained by calculating the weight of final sample minus that of the bare substrate. The weight of active material is in good agreement with the inductively coupled plasma (ICP) result of silicon element: 160 μg (i.e. 0.64 μg/mL in 250 mL) based on a sample geometry of 5 × 10 mm^2^. The Al-oxide coating in the final sample is also confirmed by aluminum (element) ICP measurement. The weight of Al based on the same sample geometry is 3.75 μg (i.e. 0.15 μg/mL in 25 mL). In view of a potential Al-contamination in Ti foil substrate, the coating Al content is estimated to be less than 2 wt.% of the framework Si content.

### Material characterization

To investigate the morphology evolution of porous Si framework thick film samples, their cross-sections and surfaces at different annealing/etching stages were observed in a focused ion beam (FIB) microscope (FEI FIB200). The surface morphology and microstructure of samples were also investigated by scanning electron microscopy (SEM, Carl Zeiss Leo 1530 VP Gemini microscope). X-ray diffraction (XRD) measurements of the samples at different annealing/etching stages were carried out by using a Philps X´Pert MRD Pro diffractometer (Cu K_α_ x-ray source, λ = 1.54 Å). The chemical compositional distributions of the annealed specimen were investigated by Auger-electron spectroscopy (AES) depth profiling and local analysis in a scanning Auger microscope (JEOL JAMP-7830F). The AES concentration depth profiles of the specimen were measured by sputtering the surface with 2 keV Ar^+^ ions in the so-called discontinuous mode. The sputtered and analyzed areas were 100 × 100 μm^2^ and 10 × 10 μm^2^ respectively. A focused 10 keV electron probe of 20 nm spot size was used to determine the local composition at the regions of interest. High-resolution transmission electron microscopy (HRTEM) investigation was performed at a JEOL 4000FX microscope at 400 kV. Core-loss electron energy-loss spectra (EELS) were acquired at Zeiss EM912 Omega microscope operated at 120 kV. The component analysis of surface and bulk by EELS at low-loss region was carried out at the sub-electronvolt sub-angstrom microscope “SESAM” (Company Carl Zeiss, Germany) with an accelerating voltage of 200 kV. The SESAM is equipped with a field-emission gun, a symmetric electrostatic Omega-type electron monochromator, and an in-column MANDOLINE energy filter. The element map was imaged with the energy-filtering transmission electron microscopy (EFTEM). A series of energy-filtered images were recorded in the low-loss range from 14 to 30 eV using a slit size of 0.45 eV. The acquisition time per frame was 10 s with CCD binning 2. The specimen drift in the image series was corrected by using the script “statistically determined spatial drift correction” (SDSD drift correction)^35^. By using the Non-Linear Least Squares Fitting (NLLS) technique, the spatial peak position of spectrum from every pixel could be determined. Consequently, different phases could be distinguished from their specific peak positions and be defined with various colors. In this way a distribution of the specific phases can be calculated as a color indexed map.

### Electrochemical test

Two-electrode Swagelok-type cells were assembled with porous Si framework thick films as working electrodes and high-purity lithium foil (Aldrich) as counter electrode. Glass fiber (GF/D) from Whatman was employed as the separator. 1 M LiPF_6_ in a non-aqueous mixture of ethylene carbonate (EC) and dimethyl carbonate (DMC) with a volume ratio of 1:1 (Ube Industries Ltd) was used as electrolyte. The cells were assembled in an Ar-filled glove box. Charge-discharge measurements were performed at room temperature under different rates from 0.1 C to 6.5 C (1 C denotes the current density to theoretically achieve multi-electron reaction to form Li_22_Si_5_ phase within 1 h) in a voltage range of 0.01-1.5 V on an Arbin MSTAT battery test system. The morphology of cycled Si frameworks after long-term 400 cycles was investigated by scanning electron microscope (SEM, Magellan 400L, FEI).

## Author Contributions

F.Q. and C.L. conceived the experiments. F.Q., Z.W., Y.W., G.R. and C.L. performed the characterizations and measurements. C.L. and F.Q. wrote the manuscript with the scientific discussions by Z.W. and H.S.

## Additional Information

**How to cite this article**: Qu, F. *et al*. Eutectic Nano-Droplet Template Injection into Bulk Silicon to Construct Porous Frameworks with Concomitant Conformal Coating as Anodes for Li-Ion Batteries. *Sci. Rep.* **5**, 10381; doi: 10.1038/srep10381 (2015).

## Supplementary Material

Supplementary Information
