# Supplementary material for: Eutectic Nano-Droplet Template Injection into Bulk Silicon to Construct Porous Frameworks with Concomitant Conformal Coating as Anodes for Li-Ion Batteries
Source: Sci Rep. 2015 May 19;5:10381. doi: 10.1038/srep10381 (PMC4437372; doi:10.1038/srep10381)
Supplement: Supplementary Information [file srep10381-s1.pdf]

Supporting Information

Eutectic Nano-Droplet Template Injection into Bulk Silicon to Construct Porous Frameworks with Concomitant Conformal Coating as Anodes for Li-Ion Batteries

Fei Qu,[2] Chilin Li,*[,1] Zumin Wang,*[,3] Yuren Wen,[3] Gunther Richter[3] & Horst P. Strunk[2]

[1]State Key Laboratory of High Performance Ceramics and Superfine Microstructure, Shanghai Institute of Ceramics, Chinese Academy of Sciences, Shanghai 200050, China. [2]Institute for Materials Science, Chair of Materials Physics, University of Stuttgart, Heisenbergstr. 3, 70569 Stuttgart, Germany. [3]Max Planck Institute for Intelligent Systems (formerly Max Planck Institute for Metals Research), Heisenbergstr. 3, 70569 Stuttgart, Germany

* E-mail: chilinli@mail.sic.ac.cn, z.wang@is.mpg.de

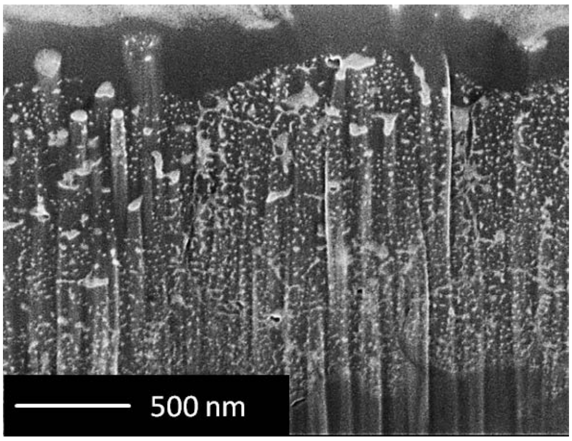

Figure S1. Magnified cross-sectional FIB image of the annealed Al/Si bilayer sample with a thickness ratio of Si:Al = 10:1. Upper layer: continuous exchanged crystalline Si (c-Si), lower layer: eutectic Al-Si droplets in bright area and c-Si in dark area.

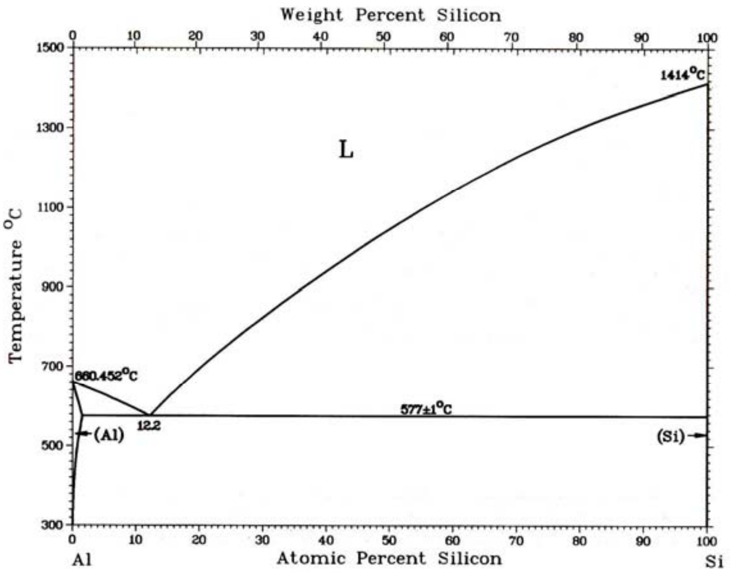

Figure S2. Al-Si phase diagram (see Ref. "A. J. McAlister, J. L. Murray, *Binary Alloy Phase Diagrams vol. 1: William W. Scott, Jr.* **1990**.")

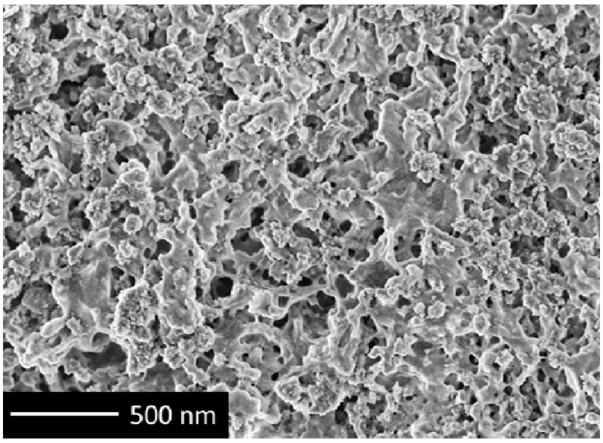

Figure S3. SEM image of cycled Si porous framework after long-term 400 cycles of charging and discharging. It is found that the nano-porosity and interconnectivity of framework are still well preserved.

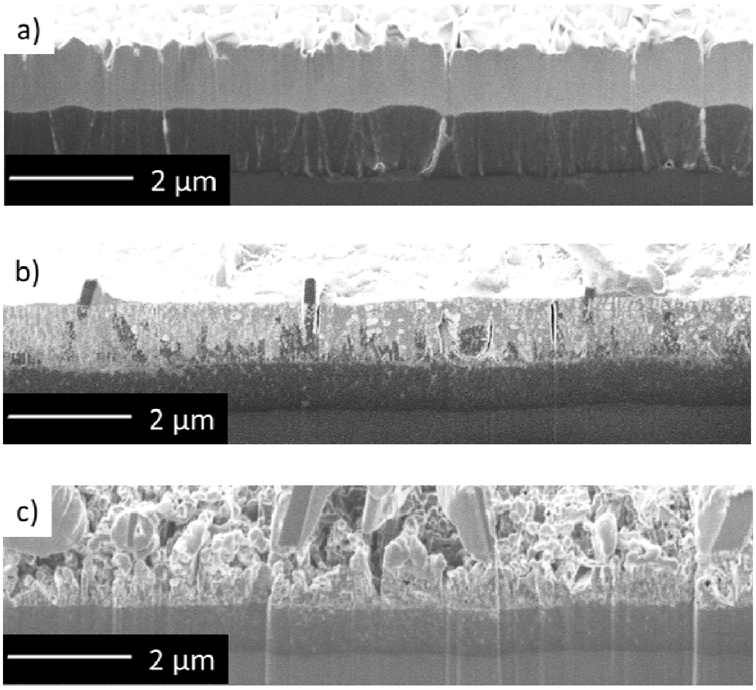

Figure S4. Cross-sectional FIB images of microstructure evolution of Al/Si bilayer samples with a thickness ratio of Si:Al = 1:1 in different fabrication stages: (a) before annealing, (b) after annealing but without etching, and (c) after additional Al etching. Note that the layer exchange between Al and Si is initiated by an Al-induced crystallization mechanism at the early annealing stage as shown in (b). However, most of Al remains at the top of specimen after annealing, meanwhile the lower part of the bottom Si layer with a thickness of ~1 µm is still compact.

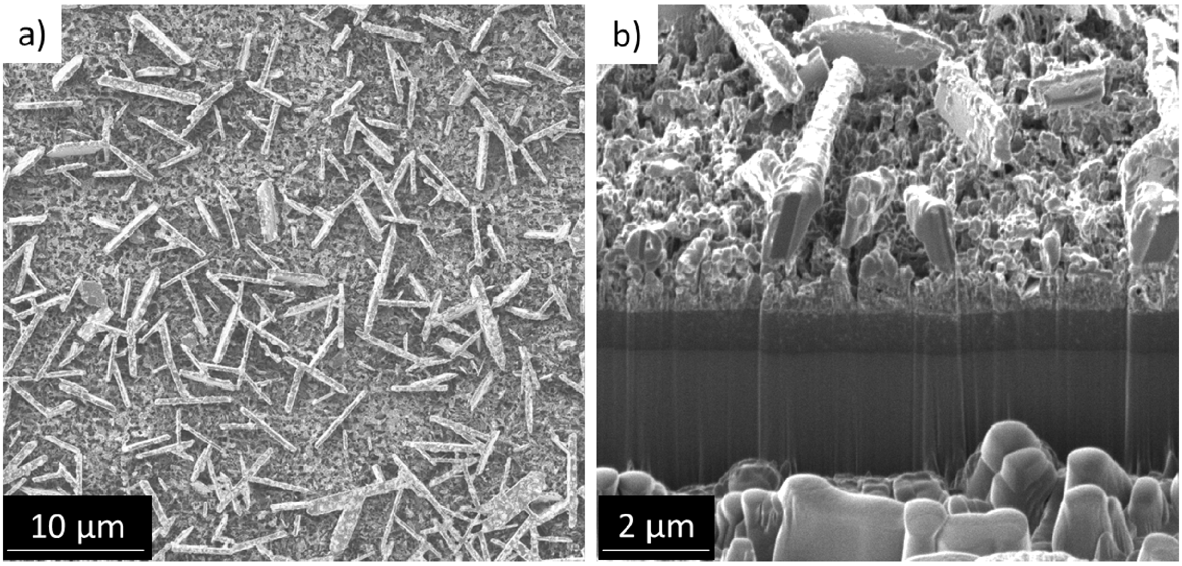

Figure S5. FIB images of the sample after annealing/cooling/etching steps: (a) top view and (b) cross-sectional view. One can find an extremely uneven surface morphology with many sheet-shaped Si of micro-sized (~5 μm) width standing irregularly over the porous upper layer of Si.
